# Patterns and Gaps Identified in a Systematic Review of the Hepatitis C Virus Care Continuum in Studies among People Who Use Drugs

**DOI:** 10.3389/fpubh.2017.00348

**Published:** 2017-12-18

**Authors:** Ashly E. Jordan, David C. Perlman, Jennifer Reed, Daniel J. Smith, Holly Hagan

**Affiliations:** ^1^Rory Meyers College of Nursing, New York University, New York, NY, United States; ^2^Center for Drug Use and HIV Research New York, New York, NY, United States; ^3^Icahn School of Medicine, Mount Sinai Beth Israel, New York, NY, United States

**Keywords:** people who use drugs, hepatitis C virus infection, hepatitis C virus care continuum, systematic review, evidence base

## Abstract

**Introduction:**

Systematic reviews are useful for synthesizing data on various health conditions and for identifying gaps in available data. In the US, the main risk group for hepatitis C virus (HCV) infection is people who use drugs (PWUD); as a group, PWUD have the highest prevalence of chronic HCV. While the care continuum construct has been increasingly applied to studies of HCV care among PWUD, what constitutes the steps in an HCV care continuum is not standardized. We sought to examine the range of HCV care continuum outcomes that studies reported on, to identify gaps in the literature, and to develop strategies that allowed for valuable syntheses of care continuum data.

**Methods:**

We conducted searches of electronic databases for published literature. Reports were eligible if they provided original data from 1990 to 2016 from the US, presented data on one or more HCV care continuum outcomes, and provided outcome data on PWUD as a distinct group.

**Results:**

A total of 313 full-text reports were assessed for eligibility. Of 212 potentially eligible reports, 32 (15.1%) did not present outcomes for PWUD separately from those who were non-PWUD. Among 101 eligible reports, a total of 166 care continuum outcomes were extracted; outcomes could be grouped into three categories that represent the HCV care continuum: testing (39.8%, *n* = 66/166); linkage to care (16.9%, *n* = 28/166); and treatment (43.4%, *n* = 72/166). Seventy-four reports (73.3%, *n* = 74/101) presented data on only one step. Linkage to care occurred variably after only antibody, or after antibody and viral load (VL) testing. Six (5.9%, *n* = 6/101) reports presented data on all three steps.

**Conclusion:**

Reports examined a variety of HCV care continuum outcomes that could be grouped into the three steps of testing, linkage to care, and treatment. The application of this care continuum model would facilitate subsequent data synthesis for program comparison and public health evaluation. Given the two-step nature of HCV testing, analyses also need to account for variation in whether linkage to care occurred after antibody testing or after sequential antibody and VL testing. Additional data are needed on the progression of PWUD through the entire care continuum.

## Introduction

Increasingly, systematic reviews (SRs), which provide a synthesis of the data in the available literature, are being used to inform public health policy, the allocation of resources, and efforts to improve population health (1–3). The process of systematically reviewing the published literature can also yield valuable insights with respect to identifying the data that do not exist in the literature, thus underscoring important research gaps and future research needs (4–7).

While the diagnosis, management, and treatment for hepatitis C virus (HCV) infection have improved over recent years, gaps in the continuity of care for HCV infection (the “HCV care continuum”) persist among people who use drugs (PWUD) (8–10). Undetected and untreated HCV infections acquired from illicit drug use (including drug injection) lead to complications that contribute to substantial global morbidity and mortality (11, 12). However, if PWUD were to successfully complete all of the steps in the care continuum, they would be effectively cured, due to the availability of highly efficacious HCV therapeutic agents. Consequently, an individual achieving a sustained virologic response (SVR) would have a reduced risk of HCV-attributable morbidity and mortality, and that individual would be rendered non-infectious. This would decrease the risk of forward transmission at both the individual and population levels. Alternatively, PWUD may be lost at any given step of the care continuum; such PWUD would remain both infectious, with the potential to contribute to ongoing transmission if they engage in risk behaviors, and at risk of HCV-attributable morbidity and mortality (1, 13).

Care continuum models, first applied to tuberculosis and sexually transmitted infections, and now formally applied as part of public health evaluation systems for HIV (14, 15), are increasingly applied to evaluate outcomes of clinical and public health efforts to control HCV infection (10). They are valuable tools for measuring and evaluating the net population or group-level effectiveness of efficacious interventions, as well as in identifying gaps in the (usually) sequential steps of care processes, which can be addressed to improve population-level outcomes (10, 16). Therefore, care continuum models may be particularly useful in addressing the efficacy/effectiveness gap in HCV research (16–19).

There is no standardized care continuum model for HCV; that is, no authoritative or scholarly body has constructed or endorsed a guidance document that provides the methods by which HCV care continuum steps should be measured and reported. Furthermore, in the US, there is significant heterogeneity in HCV testing and linkage to care methods as a function of setting, funding, and geographic location. HCV testing is a two-step process: antibody testing followed by viral load (VL) testing. In some settings and systems, both tests are performed, and those individuals with proven active infection are referred for further evaluation; however, in other settings, only antibody testing is performed, and those with positive antibodies are referred to another site for VL testing and further evaluation (20). There is also variation in the literature with respect to steps involving HCV clinical evaluation and treatment, as diagnostic tests, staging tests, and treatments have evolved (21). Previously, liver biopsy was considered a critical next step in assessing treatment eligibility; this step has largely been replaced by the use of non-invasive fibrosis measures (22, 23).

Published literature and programs have addressed progression through the entire sequence of the care continuum (i.e., reporting progression from screening through HCV treatment and SVR) and have focused on outcomes of either a single step or multiple steps of the care continuum (e.g., receipt of HCV antibody screening and, for those who are antibody positive, subsequent VL testing) (24–26).

Our SR aimed to systematically search the published scientific literature for reports that provided quantitative outcomes for PWUD moving through any one step or combinations of steps in the HCV care continuum (27). The rationale underlying this study is that PWUD as a “population” have distinct characteristics impacting outcomes and, therefore, require analysis as a separate group (28).

We sought to characterize the ways that care continuum models were applied to HCV and to identify continuum steps in the published literature that allow for valuable syntheses of data. In this paper, we present the results of the literature search and the eligibility assessments of the retrieved reports, and we highlight critical gaps in the existing research. We also present data from the SR addressing several domains that emerged as significant gaps and issues in the published literature.

## Materials and Methods

**Figure 1:**
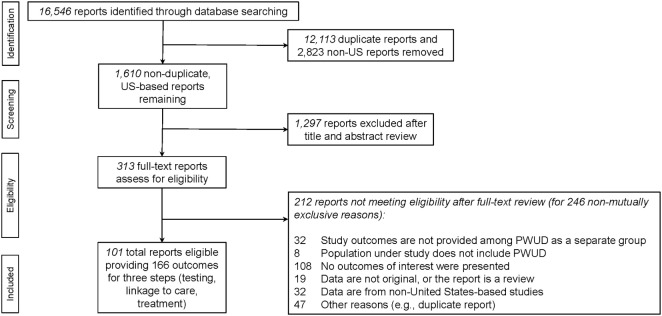
PRISMA flow diagram of hepatitis C virus care continuum systematic review.

This SR is registered with PROSPERO (#CRD42016034113); the protocol, which includes a detailed description of the study’s methods and was developed in accordance with the Preferred Reporting Items for Systematic Review and Meta-Analysis Protocols (PRISMA-P) guidelines, has been published elsewhere (27–29). As recommended by PROSPERO and using the PRISMA flow diagram, we presented the results of the SR, depicting the number of reports identified, screened, assessed for eligibility, and included (29–31); this is presented in Figure 1. We conducted electronic searches of medical literature for reports that presented original research data on the HCV care continuum among PWUD; the databases of published literature that were searched included PubMed, Ovid Embase, PsycINFO, and Cumulative Index of Nursing and Allied Health Literature. The search strategy was developed in coordination with a medical librarian at New York University. In brief, the search strategy included the intersection of the terms PWUD (and alternate phrases searching for illicit drug use such as “persons who use drugs,” “intravenous drug users,” “illicit drug use,” “substance use,” “substance abuse,” “substance dependence” as well as the abbreviations “IVDU,” “IDU,” and “PWID”), HCV (and other related terms including “Hepatitis C,” “non-A non-B hepatitis”), and other relevant key words related to the HCV care continuum (including “care coordination,” “coordination of care,” “continuum of care,” “care continuum,” “treatment retention,” “retention in care,” “engagement in care,” “treatment completion,” “treatment adherence,” “treatment initiation,” “treatment willingness,” “treatment uptake” and “care cascade”). The entire search strategy is available as an additional file to the published protocol manuscript (27).

To be eligible, the reports needed to:

(i) present outcomes of interest on any one or more of pre-specified steps in the HCV care continuum (*a priori* steps, delineated below, were chosen based on what we anticipated to find in the published literature);
(ii) provide laboratory confirmed HCV infection for the HCV diagnosis marker used in the study (antibody or VL);
(iii) present data from the US;
(iv) be published in English between 1/1/1990 and 2/20/2016;
(v) include separately reported data on PWUD and/or people who inject drugs (PWID).

Because of the variance in health-care systems internationally and, consequently, in the delivery of the steps of HCV care, which are affected by country-specific requirements and processes, we only examined data from US-based studies. Since our criteria established that eligible reports provide original data, we also excluded reports that were SRs or used simulated data; likewise, we excluded reports that relied on self-reported HCV testing data.

Two research assistants (RAs), both with graduate training in epidemiologic research, SR methodology, and biostatistics, performed the literature search and identified eligible reports. Eligibility screening and coding pilots were conducted to evaluate inter-coder reliability and refine the protocols governing report eligibility and data extraction. The RAs independently reviewed and coded all literature, which was subsequently evaluated by the research team. The research team resolved any issues that emerged throughout the study selection and data extraction processes.

The PRISMA flow diagram, which delineates the process of eligible report ascertainment, is depicted in Figure 1 (29–31). The SR began with a total of 16,546 potentially eligible reports retrieved from the primary search (see Figure 1). After removing duplicates and reports presenting data from non-United States settings, a total of 1,610 unique reports remained and were assessed for eligibility. Ultimately, 313 reports required review of their full-text to determine their eligibility for the study. Reasons for exclusion of potentially eligible reports can be found in Figure 1. We reviewed the reasons for exclusion to identify potentially addressable gaps in the literature.

Initial pre-specified plans were to examine and categorize reports and outcomes with respect to the HCV care continuum steps of (a) screening and testing, (b) linkage to and completion of clinical evaluations in care, (c) interventions to increase treatment acceptance, initiation, adherence, (d) completion of treatment, (e) achieving SVR, and (f) re-infection post-SVR (27). Among the eligible reports, all of which presented on HCV care continuum outcomes, there was variance in the definitions of continuum steps. During pilots of both eligibility screening and coding, the research team assessed the feasibility and appropriateness of applying the pre-specified HCV care continuum steps in response to what the published literature measured and reported on.

Binomial confidence intervals (CIs) were calculated for selected proportions using StatCalc, and Chi-squared tests (with corresponding *p*-values) were performed to determine statistically significant differences in the number of reports or codable outcomes per care continuum step (32).

## Results

A total of 313 full-text reports were assessed for eligibility for the SR and the set of data which could be pooled. Two hundred twelve reports did not meet inclusion criteria for the SR (see PRISMA flow diagram Figure 1). Of these, 32 reports (15.1%) were ineligible despite including PWUD in their study populations because they did not present outcomes for PWUD separately from non-PWUD.

Thus, this SR formally abstracted data from a total of 101 eligible reports yielding 166 outcomes (i.e., some reports presented data on more than one step in the HCV care continuum, hence the total number of outcomes exceeds the total number of reports). The steps addressed in the eligible reports could be readily sorted into three care continuum steps: (1) HCV testing (e.g., HCV antibody test receipt or HCV VL test receipt), (2) HCV linkage to care (e.g., referral for follow-up HCV evaluation after antibody or antibody and VL testing), and (3) HCV treatment (i.e., HCV treatment acceptance, initiation, adherence, or completion, SVR, re-infection post-SVR) (14). In order to facilitate data reduction, we grouped the extracted data into these three categories, or care continuum steps.

Each outcome was categorized into one of the three HCV care continuum steps: 66 outcomes from 42 reports on HCV testing (39.8%); 28 outcomes from 23 reports on HCV linkage to care (16.9%); and 72 outcomes from 69 reports on HCV treatment (43.4%; see Table 1).

**Table 1:** Hepatitis C virus (HCV) care continuum step outcomes presented in reports included in the systematic review (SR).

Reports and outcomes	*n* (%)	95% Binomial confidence intervals
Total number of reports included in the SR	101	Not applicable
Total number of outcomes included in the data synthesis	166	Not applicable
**HCV testing**		
Total testing reports	42/101 (41.6)	31.9–51.8%
Total testing outcomes	66/166 (39.8)	32.2–47.6%
HCV antibody testing	42/66 (63.6)	50.9–75.1%
HCV viral load (VL) testing	20/66 (30.3)	19.6–42.9%
Re-infection after spontaneous viral clearance	4/66 (6.0)	1.7–14.8%
**HCV linkage to care**		
Total linkage to care reports	23/101 (22.7)	15.0–32.2%
Total linkage to care outcomes	28/166 (16.9)	11.5–23.5%
Linkage for initial HCV VL testing	4/28 (14.3)	4.0–32.7%
All other linkage to care outcomes	24/28 (85.7)	68.5–94.3%
**HCV treatment**		
Total treatment reports	69/101 (68.3)	58.3–77.2%
Total treatment outcomes	72/166 (43.4)	35.7–51.3%
All treatment outcomes	69/72 (95.8)	0.9–11.7%
Re-infection after sustained virologic response	3/72 (4.2)	8.7–11.7%
**Reports presenting on only one step**		
Total reports presenting on a single outcome	74/101 (73.3)	63.5–81.6%
Testing only	24/101 (23.7)	15.9–33.3%
Linkage to care only	3/101 (3.0)	0.6–8.4%
Treatment only	47/101 (46.5)	36.6–56.7%
**Reports presenting on two steps and the sequence of steps reported**		
Total reports presenting on two steps	21/101 (20.8)	13.4–30.0%
Testing and linkage to care	5/101 (5.0)	1.6–11.2%
Testing and treatment	7/101 (6.9)	2.8–13.8%
Linkage to care and treatment	9/101 (8.9)	4.2–16.2%
**Reports presenting on the three steps of the HCV care continuum**		
Total reports presenting on three steps (testing, linkage to care, treatment)	6/101 (5.9)	2.2–12.5%

Among the 42 reports that presented 66 outcomes on the HCV care continuum step of testing, these 66 were classified into three categories: 4 reported on re-infection after spontaneous viral clearance, 42 reported on HCV antibody outcomes, and 20 reported on HCV VL outcomes.

Twenty of the 42 reports (47.6%; 95% CI: 32.0–63.6%) provided data on systems in which HCV antibody testing was followed by on-site HCV VL testing, for those with positive antibody. Four reports (10%; 95% CI: 2.7–22.6%) examined systems that performed HCV antibody testing on-site and then attempted to link those with positive antibody tests to off-site HCV VL testing; two of these four reports examined systems of care that included both PWUD who received on-site HCV VL testing and PWUD who were referred for off-site HCV VL testing. Therefore, these latter 2 reports contributed estimates to the data from the 20 reports that examined systems of care that provide on-site HCV VL testing to confirm active infection. 20 of the 42 reports (47.6%) did not clearly present on how HCV antibody positive patients were handled with respect to HCV VL testing.

Twenty-three reports presented data on 28 linkage to care outcomes. These fell into two categories: (1) those examining linkage for VL testing, post-positive HCV antibody (*n* = 4) or (2) linkage for evaluation for treatment, including, in some cases, diagnostic liver biopsies, post-confirmation of active infection (*n* = 24; see Table 1).

Sixty-nine reports presented 72 outcomes on the HCV treatment continuum step, including those presenting data on treatment acceptance, initiation, adherence, completion, and SVR, either alone or in various combinations. Three reports provided data on HCV re-infection after achieving SVR through treatment; these reports were categorized as representing an HCV treatment step. Forty-seven reports presented data on treatment only and did not account for losses in subsequent care continuum steps (see Table 1).

There were significantly fewer outcomes reported for linkage to care than for either testing (16.9 vs. 39.8%, *p* < 0.01) or treatment (16.3 vs. 43.4%, *p* < 0.01). Seventy-four of 101 eligible reports (73.3%) presented codable data on precisely one care continuum step; 21 (20.8%) reports provided data on two steps; and 6 (5.9%) on three steps. Significantly more reports presented data on a single step vs. multiple steps (73.3 vs. 23.7%, *p* < 0.01). Of the 27 reports (26.7%; *n* = 27/101) presenting data on two or three steps, 7 provided data on two non-sequential steps (i.e., HCV screening and HCV treatment) without explicitly stating how study participants were linked to HCV treatment following an HCV diagnosis.

## Discussion

An important role of SRs is their ability to characterize the range and extent of the published literature, and thereby provide syntheses of the data and identify critical gaps in the research (4–6). A key finding from the careful examination of the search for eligible reports for the SR and review of reasons for exclusion (see PRISMA flow diagram Figure 1) was that despite including PWUD in the study populations, 15.1% of reports were ultimately assessed as ineligible because the reports did not present quantitative data on outcomes separately among PWUD and non-PWUD in the study. This is particularly notable because the search string included drug use-related key words chosen to identify reports presenting data on PWUD specifically. Failing to present outcomes among PWUD as a distinct population is a significant lost opportunity to generate important data that could optimally inform policy and structure both HCV care and prevention systems (33).

We identified heterogeneity in the application of the care continuum construct to HCV with respect to how steps are defined, measured, and calculated (10). In reports presenting on the HIV care continuum, a similar heterogeneity in steps has been noted (34). Partly in response, the CDC has adopted two standardized HIV care continuum models with explicit definitions of each step (14). Some have suggested that a continuum model for HCV should begin with incident HCV infection and follow through to re-infection events post-SVR, HCV-induced liver disease, or liver-related mortality (10). In much of the literature the HCV care continuum starts with HCV antibody testing and continues through the receipt of HCV VL testing, referral to HCV clinical evaluation, initiation of HCV, and achievement of SVR (35). Recognizing the need for some distinctions, we propose sorting the data into the following three care continuum steps: testing, linkage to care, and treatment.

One significant area of heterogeneity was the processes or systems for doing VL testing of those found to be HCV antibody positive. HCV antibody testing can be done by finger prick or venipuncture, with results available the same day or in a few days, respectively, and is generally an inexpensive test. HCV VL testing performed by venipuncture (but not by finger prick or oral swab testing), has a turn-around time of several days and is a more expensive test. We found that fewer than half of the studies examined programs that offered on-site VL testing following antibody testing. Applications of care continuum models to HCV and SRs of HCV care continuum outcomes will need to clearly distinguish between testing which refers to just HCV antibody testing or the combination of antibody and VL testing, and accurately reflect the type of testing at which linkage to care occurs.

The search of the literature in our SR led to 101 total eligible reports presenting 166 codable outcomes. The distribution of the outcomes along the care continuum was bimodal, in that there were many reports presenting data on the “early” step of HCV screening and testing, and on the “later” step of HCV treatment outcomes. Fewer reports presented data on the “mid-continuum” step of linkage to care, despite the four that examined linkage to care for VL testing. Linkage to care is a step that has been found in some studies to substantially contribute to gaps in the HCV care continuum and, therefore, to be a significant “bottleneck” that has reduced the net population-level impact of HCV control efforts (10, 36). The availability of highly efficacious HCV treatment cannot translate into public health-level effectiveness if PWUD are not effectively engaged in care (1, 10). Notably, in the HIV care continuum, the middle step of linkage to care and initiation of antiretroviral therapy for HIV has also emerged as a bottleneck (37, 38).

Very few reports addressed more than one step in the HCV care continuum. This is a critical weakness that limits the understanding of the progression of individuals and populations through the entire HCV care continuum. Our search retrieved only 27 reports presenting on two or three steps in the HCV care continuum, and 7 of the 27 studies reported on the two non-sequential steps of screening and treatment. This is important because HCV health outcomes (e.g., treatment completion, SVR, reductions in morbidity and mortality) depend on the completion of sequential care continuum steps. Forty-seven reports provided data on HCV treatment without accounting for losses at prior steps in the HCV care continuum; this limits our ability to make strong inferences about the potential population-level impact of new HCV treatments.

Another gap in the literature appears to be a paucity of reports that either (a) identify re-infection after spontaneous clearance or (b) assess rates of re-infection post-SVR after successful treatment. Reasons for these gaps likely relate to the fact that the former requires serial VL testing of individuals, and the latter requires significant longitudinal follow-up. Only 3 out of the 69 reports presenting on HCV treatment offered data on re-infection post-SVR.

An important limitation of this analysis is that the search focused on reports that explicitly included mention of drug use or PWUD in the title or abstract. Thus, by design, the results of our search may have led to an underestimation of the number of HCV care continuum reports that either fail to present outcomes among PWUD as a separate population, or excluded PWUD in their sample population (including not having asked about drug use and, therefore, did not report on drug use). An important strength of our SR is that the search included multiple databases and multiple search terms. Further, the focus of the SR on the entire care continuum for all PWUD is an additional strength.

## Conclusion

Recommendations to be drawn from this report include that publications reflecting the HCV care continuum should explicitly report outcomes for PWUD. This would be valuable because even if sample sizes were small, pooled analyses can increase power and precision and pooled data can lead to stronger inferences that inform policy, as well as HCV care and prevention for PWUD. Data on HCV testing and linkage should clearly reflect the two-step nature of HCV testing, differentiate between “testing” that refers only to antibody testing and between “testing” that includes both antibody and VL testing, and clearly indicate the step at which linkage to care occurs. The relative paucity of codable data on the step of HCV linkage to care, as well as on programs and interventions examining the entire sequence of steps, suggests that more data are needed on the progression of PWUD through the full HCV care continuum. There is a great need to ensure effective linkages to care and to realistically assess the population-level effect of the new treatments for HCV. Reports should present data specifically for PWUD, on losses prior to treatment initiation, and on rates of spontaneous clearance and re-infection post-SVR. Additional individual- and population-level data on the progression of PWUD through the entire HCV care continuum will provide a stronger basis for public health planning for HCV control.

## Author Contributions

AJ, DP, and HH conceived the project and study design. All authors participated in the development of the study protocol. AJ and JR conducted the search and eligibility screening. AJ synthesized the results of the search and reasons for exclusion. AJ prepared the first draft of the manuscript and AJ and DP revised the manuscript with input from coauthors. All authors reviewed and approved the final submitted manuscript.

## Conflict of Interest Statement

The authors declare that the research was conducted in the absence of any commercial or financial relationships that could be construed as a potential conflict of interest.
